# Impact of peripheral lymphocyte subsets on prognosis for patients after acute ischemic stroke: A potential disease prediction model approach

**DOI:** 10.1111/cns.70023

**Published:** 2024-08-28

**Authors:** Xin Zhou, Song Xue, Xiang‐Kun Si, Wen‐Yu Du, Ya‐Nan Guo, Yang Qu, Zhen‐Ni Guo, Xin Sun

**Affiliations:** ^1^ Department of Neurology, Stroke Center the First Hospital of Jilin University Changchun China; ^2^ Department of Neurology, Neuroscience Research Center the First Hospital of Jilin University Changchun China

**Keywords:** acute ischemic stroke, immune cells, lymphocyte subsets, NIHSS, prognosis

## Abstract

**Aims:**

To investigate the relationship between peripheral blood lymphocyte subsets and prognosis in patients with acute ischemic stroke (AIS).

**Methods:**

We enrolled 294 patients with AIS and collected peripheral blood samples for analysis of lymphocyte subsets. Prognosis was assessed at 3 months using the modified Rankin Scale (mRS). Association between lymphocyte count and poor outcomes (mRS score >2) was assessed using logistic regression. Individualized prediction models were developed to predict poor outcomes.

**Results:**

Patients in the mRS score ≤2 group had higher T‐cell percentage (odds ratio [OR] = 0.947; 95% confidence interval [CI]: 0.899–0.998; *p* = 0.040), CD3^+^ T‐cell count (OR = 0.999; 95% CI: 0.998–1.000; *p* = 0.018), and CD4^+^ T‐cell count (OR = 0.998; 95% CI: 0.997–1.000; *p* = 0.030) than those in the mRS score >2 group 1–3 days after stroke. The prediction model for poor prognosis based on the CD4^+^ T‐cell count showed good discrimination (area under the curve of 0.844), calibration (*p* > 0.05), and clinical utility.

**Conclusion:**

Lower T cell percentage, CD3^+^, and CD4^+^ T‐cell counts 1–3 days after stroke were independently associated with increased risk of poor prognosis. Individualized predictive model of poor prognosis based on CD4^+^ T‐cell count have good accuracy and may predict disease prognosis.

## Graphical abstract

This analysis demonstrates that peripheral T lymphocyte counts in patients with acute ischemic stroke (AIS) are negatively correlated with the 3‐month prognosis. The correlation analysis, stratified by onset days, indicates that this conclusion is most significant in the 1–3 days post‐stroke group. Furthermore, an individualized predictive model based on CD4^+^ T‐cell count shows high accuracy, suggesting its potential as a target for interventions to improve the prognosis of patients with AIS.

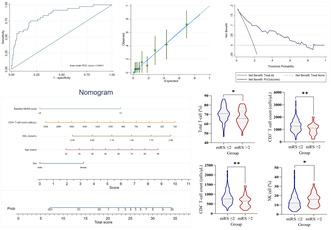

## 1 BACKGROUND

Stroke is one of the most prevalent and debilitating diseases globally. From 1990 to 2021, stroke prevalence surged by 86.1%, accompanied by a 44.1% rise in stroke‐related mortality, thereby imposing significant economic strains on individuals, families, and societies.^1^ In recent years, advances have been made in intravenous thrombolysis, cerebrovascular intervention, and conservative pharmacological treatment, yielding benefits for selected patients with acute ischemic stroke (AIS).^2^, ^3^ Nonetheless, there is a current dearth of clinical methods capable of determining early‐stage prognosis. Hence, the identification of reliable biomarkers for early prognostic prediction in acute stroke is imperative to enhance patient care and outcomes.

Mounting evidence underscores the essential involvement of immune mechanisms in ischemic stroke.^4^ Bidirectional communication between neuroinflammation and the peripheral immune system is robust throughout the course of CNS damage induced by ischemic stroke. In ischemic stroke, hypoxia in the brain tissue at the injury site initiates autoimmunity against CNS antigens. This induces intrinsic immune cells, including astrocytes and microglia, to release significant levels of proinflammatory cytokines, such as interleukin (IL)‐1β, IL‐6, interferon (IFN)‐γ, and tumor necrosis factor (TNF)‐α. These cytokines then stimulate peripheral immune cells such as neutrophils, monocytes, and T and B cells to cross the damaged blood–brain barrier and infiltrate the brain parenchyma, thereby intensifying neuroinflammation.^5^, ^6^ Concurrently, the substantial influx of cytokines into the bloodstream activates the peripheral immune system, leading to immunosuppression.^7^, ^8^, ^9^

Lymphocytes are key to both innate and adaptive immune responses. Specifically, their role in neuroinflammation is predominantly associated with T cells, which are classified based on surface markers as CD4^+^ (helper/inducer T, Th) cells and CD8^+^ (suppressor/cytotoxic T, Ts) cells.^10^ Studies have shown that T cells and their subsets penetrate the injured brain within the first 24 h after a stroke, participating in an immune response that regulates brain inflammation, and can persist for years.^11^, ^12^, ^13^ It not only releases proinflammatory factors but also secretes protective anti‐inflammatory factors. Thus, the dual nature of lymphocytes makes them pivotal in the injury, repair, and survival of ischemic brain tissue following a stroke.^14^ Based on existing reports, we hypothesized that T cells and their products could offer a potential intervention for neuronal protection against stroke injury.^15^ Consequently, our objective was to investigate the link between peripheral blood lymphocyte subset counts and proportions and the prognosis in patients with varying infarction durations. Furthermore, we aimed to ascertain the potential prognostic utility of disease prediction models for patients with AIS.

## 2 METHODS

### 2.1 Study population

This observational cohort study utilized prospectively collected data. Patients with AIS who visited the First Hospital of Jilin University stroke center between March and June 2023 were selected. Exclusion criteria included: (1) suffering from other serious systemic diseases such as autoimmune disorders, (2) acute infections, and (3) neurological deficits resulting from trauma or malignancy. Furthermore, clinical prognosis was evaluated utilizing the mRS score. A telephone follow‐up was conducted 90 days post‐stroke to assess the extent of neurological impairment using the mRS score. Typically, a mRS score of ≤2 was indicative of a favorable prognosis, reflecting a minimal neurological deficit, whereas a mRS score >2 was associated with a poor prognosis. This study was granted by the Ethics Committee of the First Hospital of Jilin University (22k047‐003).

### 2.2 Data collection

Data were obtained on demographic and clinical characteristics (age, sex, previous stroke, diabetes, hypertension, coronary artery disease, smoking and alcohol consumption, antihypertensive, hypoglycemic, and antiplatelet agents), laboratory tests [levels of total cholesterol, triglycerides, homocysteine, low‐density lipoprotein (LDL) cholesterol, and high‐density lipoprotein (HDL) cholesterol], the TOAST (Trial of Org 10,172 in Acute Stroke Treatment) subtype, and stroke severity as measured by the National Institutes of Health Stroke Scale (NIHSS) score on admission. Stroke severity was classified as mild (NIHSS score ≤4) and moderately severe (NIHSS score >4) according to the scale. Smoking was considered to be the daily use of at least one cigarette for a consecutive year. Alcohol consumption was characterized by the intake of one or more drinks per day over the past year. Hypertension was identified by either a recorded history or a clinical diagnosis established during the hospital stay. Diabetes was defined similarly by either a documented history or a hospital diagnosis.

### 2.3 Lymphocyte typing

For patients with AIS, blood samples were collected the next morning after hospital admission. Flow cytometry was used to identify and quantify T lymphocytes (CD19^−^CD3^+^), B lymphocytes (CD3^−^CD19^+^), natural killer (NK) cells (CD3^−^/CD16^+^CD56^+^), Th cells (CD3^+^CD4^+^), Ts cells (CD3^+^CD8^+^), and CD4^+^/CD8^+^ cells. The proportions of lymphocyte types among total leukocytes were calculated.

### 2.4 Statistical analysis

Stata 17.0 (StataCorp) and IBM SPSS Statistics 23.0 (IBM) were used for statistical analysis. Data distribution normality was evaluated with the Shapiro–Wilk test. Normally distributed continuous variables are expressed as mean ± SD and compared via the Student *t* test for independent samples. Non‐normally distributed variables are reported as median (interquartile range) and analyzed with the Mann–Whitney *U* test. Categorical variables are presented as frequencies, with between‐group differences assessed using the χ^2^ test. Univariate and multivariate logistic regression analyses were performed to examine the association of lymphocyte subsets with outcomes. Model 1 had no adjustments, Model 2 included adjustments for sex and age, and Model 3 included adjustments for all variables, with *p* < 0.05.

Individualized prognostic models for adverse outcomes were constructed using multivariate logistic regression analysis. Backward elimination based on the Akaike Information Criterion (AIC) was used for variable selection. A nomogram was then developed by Stata 17.0, and internal validation was conducted by bootstrapping with 1000 bootstrap samples. Model discrimination was evaluated using the area under the receiver operating characteristic curve (AUC‐ROC), and goodness of fit was assessed with the Hosmer–Lemeshow test. Model consistency and accuracy were further examined through calibration curves. Decision curve analysis (DCA) was employed to quantify the net benefit and determine the clinical utility of the nomogram.^16^ All tests were two‐sided, with statistical significance defined as *p* < 0.05.

## 3 RESULTS

### 3.1 Baseline characteristics of the patients

This study initially enrolled 354 patients with AIS based on the specified inclusion criteria, with 294 patients included in the final analysis. Detailed baseline clinical features of the patients are listed in Table 1.

**TABLE 1:** Baseline characteristics of patients according to mRS classification.

	Total	Favorable outcome (mRS ≤2)	Poor outcome (mRS >2)	Test value	*p*
*N*, *n* (%)	294	239 (81.3)	55 (18.7)		
Age, years, median (IQR)	61.5 (53–71)	61 (52–69)	66 (58–76)	−3.296	0.001
*Sex*					
Male, *n* (%)	210 (71.4)	180 (75.3)	30 (54.5)	9.450	0.002
Female, *n* (%)	84 (28.6)	59 (24.7)	25 (45.5)		
Alcohol consumption, *n* (%)	82 (27.9)	71 (29.7)	11 (20)	2.095	0.148
Smoking, *n* (%)	107 (36.4)	91 (38.1)	15 (27.3)	2.263	0.132
Coronary artery disease, *n* (%)	27 (9.2)	19 (7.9)	8 (14.5)	2.332	0.127
Hypertension, *n* (%)	177 (60.2)	141 (60.0)	36 (65.5)	0.778	0.378
Diabetes, *n* (%)	98 (33.3)	76 (31.8)	22 (40.0)	1.353	0.245
Previous stroke, *n* (%)	70 (23.8)	50 (20.9)	20 (36.4)	5.878	0.015
Antihypertensive drugs, *n* (%)	156 (53.1)	123 (51.5)	33 (60.0)	1.308	0.253
Hypoglycemic agents, *n* (%)	90 (30.6)	69 (28.9)	21 (38.2)	1.825	0.177
Antiplatelet agents, *n* (%)	65 (22.1)	48 (20.1)	17 (30.9)	3.043	0.081
*Baseline NIHSS score*					
Baseline NIHSS score, median (IQR)	2 (0–5)	1 (0–3)	6 (2–10)	−7.543	<0.001
NIHSS ≤4, *n* (%)	219 (73.0)	198 (81.8)	21 (36.2)	49.383	<0.001
NIHSS >4, *n* (%)	81 (27.0)	44 (18.2)	37 (63.8)		
Homocysteine, μmol/L, median (IQR)	13.3 (10.7–17.8)	13.0 (10.4–16.9)	14.6 (12.1–19.4)	−2.280	0.006
Triglyceride, mmol/L, median (IQR)	1.49 (1.07–2.07)	1.49 (1.07–2.10)	1.49 (1.07–1.99)	−0.343	0.732
Total cholesterol, mmol/L, median (IQR)	4.59 (3.87–5.29)	4.56 (3.86–5.15)	4.94 (3.89–5.66)	−1.650	0.099
HDL, mmol/L, median (IQR)	1.02 (0.89–1.20)	0.99 (0.89–1.16)	1.16 (0.95–1.31)	−3.025	0.002
LDL, mmol/L, mean ± SD	2.94 ± 0.80	2.90 ± 0.76	3.12 ± 0.94	−2.071	0.039
*TOAST*					
Large‐artery atherosclerosis, *n* (%)	149 (50.7)	121 (50.7)	28 (50.9)	3.522	0.172
Small‐vessel occlusion, *n* (%)	128 (43.5)	107 (44.8)	21 (38.2)		
Cardioembolism, *n* (%)	8 (2.7)	7 (2.9)	1 (1.8)		
The other types, *n* (%)	9 (3.1)	7 (2.9)	2 (3.6)		
*Cell types*					
Total T‐cell, %, median (IQR)	71.0 (65.5–76.0)	71.2 (66.0–76.0)	68.7 (62.2–75.1)	−0.031	0.074
CD3^+^ T‐cell count, cells/μL, median (IQR)	1229 (930–1571)	1272 (975–1615)	1111 (694–1353)	−0.001	0.001
Th cell, %, mean ± SD	44.0 ± 8.8	44.5 ± 8.5	42.2 ± 9.9	1.727	0.085
CD4^+^ T‐cell count, cells/μL, median (IQR)	749.5 (553.3–1012.8)	776 (586–1039)	677 (373–799)	−0.002	0.001
Ts cell, %, median (IQR)	22.4 (17.3–27.5)	22.4 (17.5–27.3)	22.2 (15.7–30.7)	−0.002	0.927
CD8^+^ T‐cell count, cells/μL, median (IQR)	386 (272–520)	391 (290–526)	362 (210–469)	−0.002	0.019
CD4^+^/CD8^+^, median (IQR)	1.98 (1.45–2.78)	1.96 (1.46–2.77)	2.03 (1.34–3.03)	−0.043	0.772
B‐cell, %, median (IQR)	14.2 (10.68–18.4)	14.0 (10.5–18.4)	14.4 (11.0–19.1)	−0.009	0.549
CD19^+^ B‐cell count, cells/μL, median (IQR)	223 (153–341)	231 (156–356)	204 (120–315)	−0.002	0.038
NK cell, %, median (IQR)	11.5 (7.9–17.9)	11.4 (7.6–16.2)	13.4 (9.0–18.7)	0.031	0.078
CD16^+^CD56^+^ NK cell count, cells/μL, median (IQR)	195 (131–289)	197 (134–294)	192 (125–288)	−0.001	0.566

*Note*: LDL and Th cell percentages are presented as mean ± SD and evaluated using the Student *t* test. Age, homocysteine, triglyceride, total cholesterol, HDL, total T‐cell percentage, CD3^+^ T‐cell count, CD4^+^ T‐cell count, Ts cell percentage, CD8^+^ T‐cell count, CD4^+^/CD8^+^, B‐cell percentage, CD19^+^ B‐cell count, NK cell percentage, and CD16^+^CD56^+^ NK cell count are expressed as median (interquartile range) and evaluated using the Mann–Whitney *U* test. The remaining variables are shown as *n* (%) and assessed using the χ^2^ test.

Abbreviations: HDL, high‐density lipoprotein cholesterol; B‐cell percentage, B‐lymphocytes (CD3^−^CD19^+^) counts/leukocyte counts; LDL, low‐density lipoprotein cholesterol; NIHSS, National Institutes of Health Stroke Scale; NK cell percentage, Natural Killer cell (CD3^−^/CD16^+^CD56^+^) counts/leukocyte counts; Th cell percentage, helper T cells (CD3^+^CD4^+^) counts/leukocyte counts; TOAST, the Trial of Org 10,172 in Acute Stroke Treatment; Total T‐cell percentage, T‐lymphocytes (CD19^−^CD3^+^) counts/leukocyte counts; Ts cell percentage, suppressor/cytotoxic T cells (CD3^+^CD8^+^) counts/leukocyte counts.

Compared to patients in the mRS score ≤2 group, patients in the mRS score >2 group were older (median 61 versus 66 years, *p* = 0.001), had a lower proportion of males (75.3% versus 54.5%, *p* = 0.002), and had a higher proportion of patients with previous stroke (20.9% versus 36.4%, *p* = 0.015). In addition, homocysteine (median 13.0 versus 14.6 μmol/L, *p* = 0.006), HDL cholesterol (median 1.00 versus 1.11 mmol/L, *p* = 0.005), LDL cholesterol (median 2.90 versus 3.12 mmol/L, *p* = 0.039), and NIHSS score (median 1.0 versus 6.0, *p* < 0.001) were significantly higher in the mRS score >2 group than in the mRS score ≤2 group. There were no statistically significant differences between the two groups in terms of risk factors for cerebrovascular disease such as smoking, alcohol consumption, hypertension, diabetes mellitus, coronary artery disease, total cholesterol, and triglycerides.

Regarding the lymphocyte subsets, patients in the mRS score >2 group had significantly lower absolute CD3^+^ T‐cell count (median 1272 versus 1111 cells/μL, *p* = 0.001), CD4^+^ T‐cell count (median 776 versus 677 cells/μL, *p* = 0.001), CD8^+^ T‐cell count (median 391 versus 362 cells/μL, *p* = 0.019), and CD19^+^ B‐cell count (median 231 versus 204 cells/μL, *p* = 0.038) than patients in the mRS score ≤2 group. None of the 294 patients received intravenous or mechanical thrombolysis.

### 3.2 Association between the lymphocyte subsets and the severity of clinical outcomes

**FIGURE 1:**
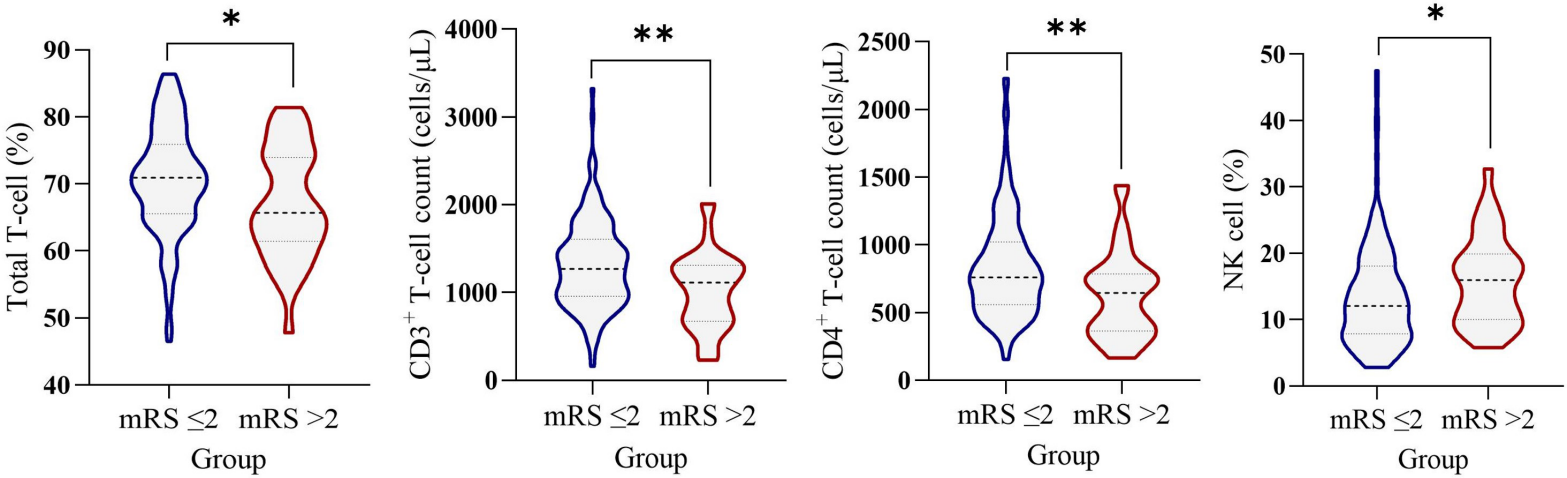
Violin plots of lymphocyte subset distributions that were significantly different between the different prognostic groups in the 1–3 days group. *p < 0.05 and **p < 0.01 by the Mann–Whitney *U* test or the Student *t* test.

Patients were categorized into three subgroups based on the interval between stroke onset and hospitalization: the 1–3, 4–7, and 8–14 days groups. The proportions and counts of lymphocyte subsets were not statistically different with respect to the outcomes in the 4–7 and 8–14 days groups (Table 2). In the 1–3 days group, total T‐cell percentage (mean 70.6 versus 67.0, *p* = 0.016), CD3^+^ T‐cell count (median 1267 versus 1112 cells/μL, *p* = 0.002), and CD4^+^ T‐cell count (median 760.0 versus 646.5 cells/μL, *p* = 0.002) were significantly higher in patients in the mRS score ≤2 group than in the mRS score >2 group. Additionally, the NK cell percentage (median 12 versus 16, *p* = 0.019) was lower in the mRS score ≤2 group than in the mRS score >2 group (Figure 1).

**TABLE 2:** Relationship between temporal changes in lymphocyte subsets and 3‐month prognosis in patients with AIS.

	1–3 days			4–7 days			8–14 days		
	mRS ≤2	mRS >2	*p*	mRS ≤2	mRS >2	*p*	mRS ≤2	mRS >2	*p*
Total T‐cell, %, mean ± SD	70.6 ± 8.0	67.0 ± 8.3	0.016	72 (67–76)	71 (64–77)	0.747	71.5 (67.0–75.7)	74.2 (68.9–81.4)	0.274
CD3^+^ T‐cell count, cells/μL, median (IQR)	1267 (957–1607)	1112 (672–1311)	0.002	1179 (977–1669)	1047 (579–1527)	0.300	1324 (1032–1611)	1051 (809–1233)	0.198
Th cell, %, mean ± SD	43.8 ± 8.6	40.9 ± 9.9	0.072	46.3 (40.7–52.0)	41.8 (31.4–52.7)	0.428	45.7 ± 8.7	48.9 ± 5.7	0.329
CD4^+^ T‐cell count, cells/μL, median (IQR)	760.0 (559.8–1022.0)	646.5 (364.8–787.0)	0.002	860.4 ± 370.4	732.8 ± 434.5	0.311	857.1 ± 298.8	714.9 ± 188.9	0.230
Ts cell, %, median (IQR)	23.4 (17.6–27.6)	21.4 (15.0–30.5)	0.582	22.3 ± 6.3	23.0 ± 7.1	0.771	20.0 (17.2–26.0)	23.0 (15.6–31.3)	0.632
CD8^+^ T‐cell count, cells/μL, median (IQR)	408 (314–526)	363 (211–497)	0.050	364 (261–554)	351 (214–494)	0.451	372 (286–526)	312 (207–421)	0.427
CD4^+^/CD8^+^, median (IQR)	1.90 (1.39–2.73)	1.99 (1.10–3.26)	0.956	2.01 (1.60–2.78)	1.92 (1.34–2.68)	0.550	2.21 (1.74–2.96)	2.10 (1.50–3.55)	0.956
B‐cell, %, median (IQR)	13.2 (9.9–18.0)	14.4 (11.0–19.4)	0.250	15.4 (12.1–18.8)	15.7 (11.8–19.4)	0.953	14.1 (10.8–19.1)	11.1 (7.70–14.0)	0.055
CD19^+^ B‐cell count, cells/μL, median (IQR)	213 (156–357)	201 (131–281)	0.208	243 (185–368)	278 (88–350)	0.807	262 (164–352)	165 (101–243)	0.082
NK cell, %, median (IQR)	12.0 (7.8–18.2)	16.0 (10.0–19.9)	0.019	9.5 (7.0–14.4)	10.2 (8.2–22.9)	0.428	11.2 (7.5–14.5)	11.9 (7.6–16.0)	0.403
CD16^+^CD56^+^ NK cell count, cells/μL, median (IQR)	200 (140–326)	193 (127–296)	0.885	186 (118–236)	182 (110–295)	0.852	206 (126–272)	181 (128–217)	0.520

*Note*: Total T‐cell percentage and Th cell percentages are expressed as mean ± SD and evaluated using the Student *t* test. CD3^+^ T‐cell count, CD4^+^ T‐cell count, Ts cell percentage, CD8^+^ T‐cell count, CD4^+^/CD8^+^, B‐cell percentage, CD19^+^ B‐cell count, NK cell percentage, and CD16^+^CD56^+^ NK cell count are expressed as median (interquartile range) and evaluated using the Mann–Whitney *U* test.

Abbreviations: AIS, acute ischemic stroke; B‐cell percentage, B‐lymphocytes (CD3^−^CD19^+^) counts/leukocyte counts; NK cell percentage, Natural Killer cell (CD3^−^/CD16^+^CD56^+^) counts/leukocyte counts; Th cell percentage, helper T cells (CD3^+^CD4^+^) counts/leukocyte counts; Total T‐cell percentage, T‐lymphocytes (CD19^−^CD3^+^) counts/leukocyte counts; Ts cell percentage, suppressor/cytotoxic T cells (CD3^+^CD8^+^) counts/leukocyte counts.

To determine whether total T‐cell percentage, CD3^+^ T‐cell count, CD4^+^ T‐cell count, and NK‐cell percentage independently predicted outcomes at 3 months among patients in the 1–3 days group, variables with *p* < 0.05 in the bivariate analysis were included in the multifactorial analysis. Table 3 shows the baseline clinical features of patients in the AIS 1–3 days group. Multifactorial regression analyses showed that higher total T‐cell percentage (OR = 0.947; 95% CI: 0.899–0.998; *p* = 0.040), CD3^+^ T‐cell count (OR = 0.999; 95% CI: 0.998–1.000; *p* = 0.018), and CD4^+^ T‐cell count (OR = 0.998; 95% CI: 0.997–1.000; *p* = 0.030) were independently associated with a favorable prognosis (Table 4).

**TABLE 3:** The baseline clinical characteristics of the patients in the AIS 1–3 days group.

	Total	Favorable outcome (mRS ≤2)	Poor outcome (mRS >2)	Test value	*p*
*N*, *n* (%)	186	150 (80.6)	36 (19.4)		
Age, years, median (IQR)	64 (54–72)	61 (53–71)	68 (59–78)	−2.935	0.003
*Sex*					
Male, *n* (%)	135 (72.6)	115 (76.7)	20 (55.6)	6.502	0.011
Female, *n* (%)	51 (27.4)	35 (23.3)	16 (44.4)		
Alcohol consumption, *n* (%)	46 (24.7)	40 (26.7)	6 (16.7)	1.560	0.212
Smoking, *n* (%)	60 (32.3)	53 (35.3)	7 (19.4)	3.354	0.067
Coronary artery disease, *n* (%)	20 (10.8)	14 (9.3)	6 (16.7)	1.627	0.201
Hypertension, *n* (%)	110 (59.1)	91 (60.7)	19 (52.8)	0.748	0.387
Diabetes, *n* (%)	57 (30.6)	44 (29.3)	13 (36.1)	0.628	0.428
Previous stroke, *n* (%)	46 (24.7)	33 (22.0)	13 (36.1)	3.106	0.078
Antihypertensive drugs, *n* (%)	94 (50.5)	76 (50.7)	18 (50.0)	0.005	0.943
Hypoglycemic agents, *n* (%)	50 (26.9)	38 (25.3)	12 (33.3)	0.943	0.331
Antiplatelet agents, *n* (%)	41 (22.0)	31 (20.7)	10 (27.8)	0.854	0.355
*Baseline NIHSS score*					
Baseline NIHSS score, median (IQR)	2 (0–5)	1 (0–4)	8 (3–11)	−5.891	<0.001
NIHSS ≤4, *n* (%)	129 (69.4)	117 (78.0)	12 (33.3)	27.253	<0.001
NIHSS >4, *n* (%)	57 (30.6)	33 (22.0)	24 (66.7)		
Homocysteine, μmol/L, median (IQR)	13.5 (10.7–18.5)	13.2 (10.4–18.1)	14.5 (12.7–22.3)	−2.280	0.023
Triglyceride, mmol/L, median (IQR)	1.50 (1.04–2.04)	1.52 (1.06–2.11)	1.32 (1.00–1.82)	−1.226	0.220
Total cholesterol, mmol/L, median (IQR)	4.75 (3.89–5.40)	4.66 (3.88–5.30)	5.03 (3.93–6.36)	−1.572	0.116
HDL, mmol/L, median (IQR)	1.03 (0.90–1.23)	1.02 (0.89–1.20)	1.19 (0.97–1.33)	−2.448	0.014
LDL, mmol/L, mean ± SD	3.05 ± 0.81	2.98 ± 0.74	3.31 ± 1.03	−2.157	0.032
*TOAST*					
Large‐artery atherosclerosis, *n* (%)	93 (50.0)	78 (52.0)	15 (41.7)	2.565	0.277
Small‐vessel occlusion, *n* (%)	78 (41.9)	62 (41.3)	16 (44.4)		
Cardioembolism, *n* (%)	8 (4.3)	7 (4.7)	1 (2.8)		
The other types, *n* (%)	7 (3.8)	6 (4.0)	1 (2.8)		
*Cell types*					
Total T‐cell, %, mean ± SD	69.9 ± 8.2	70.6 ± 8.0	67.0 ± 8.3	2.437	0.016
CD3^+^ T‐cell count, cells/μL, median (IQR)	1240 (910–1524)	1267 (957–1607)	1112 (672–1311)	−3.166	0.002
Th cell, %, mean ± SD	43.3 ± 8.9	43.8 ± 8.6	40.9 ± 9.9	1.810	0.072
CD4^+^ T‐cell count, cells/μL, median (IQR)	740.5 (528.0–970.3)	760.0 (559.8–1022.0)	646.5 (364.8–787.0)	−3.156	0.002
Ts cell, %, median (IQR)	23.2 (17.3–28.3)	23.4 (17.6–27.6)	21.4 (15.0–30.5)	−0.550	0.582
CD8^+^ T‐cell count, cells/μL, median (IQR)	397 (282–518)	408 (314–526)	363 (211–497)	−1.962	0.050
CD4^+^/CD8^+^, median (IQR)	1.90 (1.36–2.77)	1.90 (1.39–2.73)	1.99 (1.10–3.26)	−0.055	0.956
B‐cell, %, median (IQR)	13.7 (10.1–18.3)	13.2 (9.9–18.0)	14.4 (11.0–19.4)	−1.150	0.250
CD19^+^ B‐cell count, cells/μL, median (IQR)	211 (153–334)	213 (156–357)	201 (131–281)	−1.260	0.208
NK cells, %, median (IQR)	12.2 (8.2–18.4)	12.0 (7.8–18.2)	16.0 (10.0–19.9)	−2.343	0.019
CD16^+^CD56^+^ NK cell count, cells/μL, median (IQR)	197 (139–310)	200 (140–326)	193 (127–296)	−0.145	0.885

LDL, total T‐cell percentage, and Th cell percentage are presented as mean ± SD and evaluated using the Student *t* test. Age, homocysteine, triglyceride, total cholesterol, HDL, CD3^+^ T‐cell count, CD4^+^ T‐cell count, Ts cell percentage, CD8^+^ T‐cell count, CD4^+^/CD8^+^, B‐cell percentage, CD19^+^ B‐cell count, NK cell percentage, and CD16^+^CD56^+^ NK cell count are expressed as median (interquartile range) and evaluated using the Mann–Whitney *U* test. The remaining variables are shown as *n* (%) and assessed using the χ^2^ test.

Abbreviations: B‐cell percentage, B‐lymphocytes (CD3^−^CD19^+^) counts/leukocyte counts; HDL, high‐density lipoprotein cholesterol; LDL, low‐density lipoprotein cholesterol; NIHSS, National Institutes of Health Stroke Scale; NK cell percentage, Natural Killer cell (CD3^−^/CD16^+^CD56^+^) counts/leukocyte counts; Total T‐cell percentage, T‐lymphocytes (CD19^−^CD3^+^) counts/leukocyte counts; Th cell percentage, helper T cells (CD3^+^CD4^+^) counts/leukocyte counts; TOAST, the Trial of Org 10,172 in Acute Stroke Treatment; Ts cell percentage, suppressor/cytotoxic T cells (CD3^+^CD8^+^) counts/leukocyte counts.

**TABLE 4:** Univariate and multivariate logistic analysis of CD3^+^ T‐cell count, CD4^+^ T‐cell count, total T‐cell percentage, and poor 3‐month outcome of patients in the AIS 1–3 days group.

	Model 1		Model 2		Model 3	
	OR (95% CI)	*p*	OR (95% CI)	*p*	OR (95% CI)	*p*
CD3^+^ T‐cell count	0.999 (0.998–0.999)	0.002	0.998 (0.997–0.999)	0.002	0.999 (0.998–1.000)	0.018
CD4^+^ T‐cell count	0.998 (0.997–0.999)	0.003	0.998 (0.996–0.999)	0.005	0.998 (0.997–1.000)	0.030
Total T‐cell, %	0.947 (0.905–0.991)	0.018	0.946 (0.903–0.992)	0.023	0.947 (0.899–0.998)	0.040

*Note*: Model 1 was a univariate analysis. CD3^+^ T‐cell count, CD4^+^ T‐cell count, and total T‐cell percentage were associated with different outcomes at 3 months (*p* < 0.05). Model 2 was adjusted for sex and age. Model 3 was adjusted for sex, age, baseline NIHSS score, HDL, and LDL. Low CD3^+^ T‐cell count, CD4^+^ T‐cell count, and total T‐cell percentage (*p* < 0.05) were independent predictors of poor 3‐month prognosis, according to multivariate logistic regression analysis.

Abbreviations: CI, confidence interval; OR, odds ratio.

### 3.3 Individualized prediction model

**FIGURE 2:**
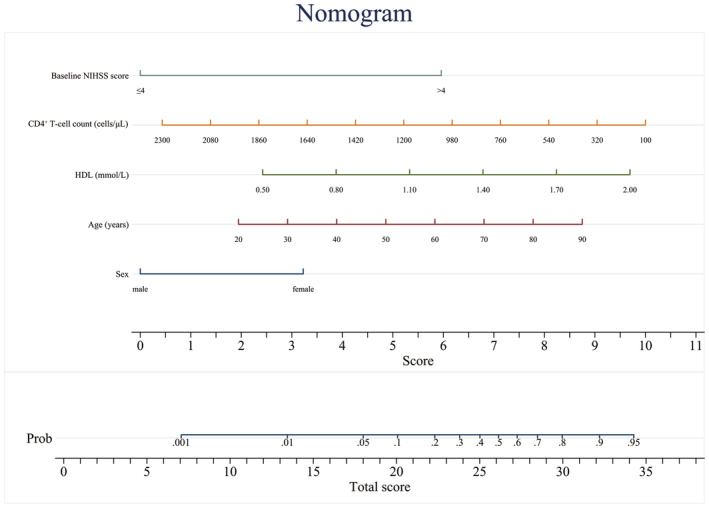
The nomogram for predicting outcomes. It was composed of two areas; the upper section calculates the total score by summing the scores assigned to each variable. Each variable's score is determined by projecting its value onto the score line and recording the corresponding score. The lower section calculates the probability (Prob) of patients experiencing an unfavorable functional outcome. HDL, high‐density lipoprotein cholesterol; NIHSS, National Institutes of Health Stroke Scale.

**FIGURE 3:**
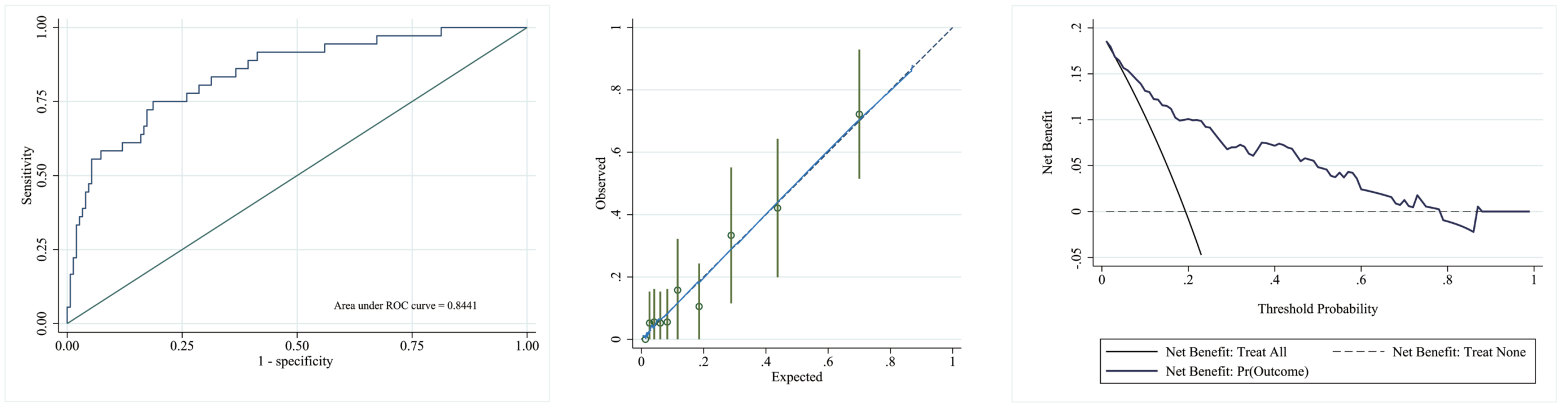
ROC curves, calibration curve, and DCA of the nomogram model to predict outcome. ROC (left), calibration curve (middle), and DCA (right). In detail, the model's discrimination is assessed using ROC analysis. The dashed line in the calibration curves with bootstraps of 1000 resamples represents the ideal reference line, and the green vertical line represents the 95% CI as measured by Hosmer–Lemeshow analysis. The calibration curves show that there is good agreement between the model predictions (*x*‐axis) and the actual observations (*y*‐axis). In DCA, the *x*‐axis denotes the risk threshold for unfavorable outcomes, while the *y*‐axis represents the net benefit across various thresholds. A more distant curve signifies a higher net benefit. The black line represents the scenario where all patients have a poor prognosis, and the gray dotted line indicates that no patients have a poor prognosis. The other line shows the net benefit as predicted by the model. DCA, decision curve analysis; ROC, receiver operating characteristic curve.

Combining the results of the multivariate logistic analyses in the 1–3 days group and using backward exclusion based on the AIC for variable selection, the final individualized prediction model for disease regression included sex (categorical variable), age (continuous variable), baseline NIHSS score (categorical variable), HDL (continuous variable), and absolute CD4^+^ T‐cell count (continuous variable). The predictive model was visualized using a nomogram (Figure 2). Internal validation was done using the self‐sampling of 1000 bootstrap samples. The model demonstrated good discrimination with an AUC‐ROC value of 0.8441 (95% CI: 0.771–0.917). The optimal cutoff point was 0.237, with a sensitivity of 75% and a specificity of 81.3%. The calibration curve indicated that the model had reliable predictive accuracy. The Hosmer–Lemeshow test yielded *p* = 0.992, implying that the model was appropriately calibrated. The DCA curve reflected a relatively favorable net clinical benefit (Figure 3).

## 4 DISCUSSION

The objective of this study was to elucidate the link between peripheral blood lymphocyte subsets and prognosis in patients with AIS. Prior research involving animal models has revealed that infarct size is reduced in mice lacking T cells, suggesting that T cell invasion into the brain may have deleterious effects.^17^, ^18^ However, in our current study, we observed an elevated percentage of circulating T cells and increased counts of CD3^+^ and CD4^+^ T‐cells in patients exhibiting favorable prognostic outcomes, consistent with findings from several preceding clinical trials.^19^, ^20^ This can be attributed to multiple underlying mechanisms.

It is well known that in instances of ischemic stroke, neuroinflammation begins within minutes of the onset of ischemia and persists for several days. This process primarily involves the infiltration of various peripheral immune cells and the activation of resident immune cells in the CNS, resulting in the release of diverse cytokines.^21^, ^22^, ^23^ During this process, microglia in the brain activate CD4^+^ T cells, prompting their differentiation into either Th type 1 (Th1) cells, which secrete proinflammatory factors, or Th type 2 (Th2) cells, which release anti‐inflammatory factors.^24^ Experimental studies in mice have demonstrated an increased differentiation of the immune response toward the Th2‐type following the acute phase of a cerebrovascular event.^25^ Therefore, we hypothesized that the elevated ratio of Th2 cells relative to Th1 cells results in increased release of anti‐inflammatory factors, potentially playing a protective role in neural recovery and contributing to improved patient outcomes. It has been demonstrated that IL‐4 plays a critical role in the differentiation of T cells into Th2 cells.^26^, ^27^ Thus, whether IL‐4 can be targeted as an intervention to mitigate neuroinflammation by balancing Th1 and Th2 cells warrants further exploration.

Existing evidence indicates that following ischemia, there is a notable upregulation in gene expression of TNF‐α, IFN‐γ, and IL‐1β in mice deficient in regulatory T (Treg) cells. Additionally, antagonizing both TNF‐α and IFN‐γ has been shown to significantly decrease infarct size.^28^ Therefore, we hypothesized that the protective role of T cells after AIS is associated with a specific T cell subset, the Treg cells. Early Tregs achieve protective immunomodulation mainly through the secretion of IL‐10.^29^ Moreover, inflammatory mediators may exert beneficial effects on stroke recovery. Activated intrinsic immune cells like astrocytes not only release damaging proinflammatory factors into the brain but also upregulate protective cytokines such as TGF‐β, IL‐33, and IL‐2. These cytokines can bind to receptors within the CNS and corresponding receptors outside the CNS, thereby facilitating the development of Treg cells in the peripheral blood and enhancing their protective effects.^28^, ^30^, ^31^

In addition to inducing neuroinflammation following an ischemic stroke, another immune cascade response known as stroke‐induced immunosuppression is triggered. Cytokines and inflammatory cells produced in the brain traverse the blood–brain barrier into the bloodstream, activating the peripheral immune system through the sympathetic nervous system and the hypothalamus‐pituitary–adrenal axis. This activation initiates a systemic inflammatory response characterized by reduced peripheral blood and splenic lymphocytes, as well as suppressed T‐cell proliferation, leading to overall immune system suppression.^32^, ^33^, ^34^ However, glucocorticoids, the end product of the hypothalamus–pituitary–adrenal axis, despite their immunosuppressive effects, also stimulate the production of anti‐inflammatory cytokines such as IL‐10. They facilitate the proportional shift in the balance from Th1 cells to Th2 cells and thereby mitigate neuroinflammation, promoting neurological recovery.^35^, ^36^ Therefore, we posit that the higher proportion of peripheral blood lymphocytes observed in patients with favorable prognoses may be linked to this mechanism, suggesting a promising avenue for future investigation.

Furthermore, we developed an individualized predictive model to predict adverse outcomes, with the model including age, sex, baseline NIHSS score, and HDL and CD4^+^ T‐cell count. The metrics demonstrated that our model exhibited robust performance. Therefore, we conclude that the model based on CD4^+^ T‐cell count has a good predictive value for patient prognosis.

Although we initially explored possible factors that may influence and improve the prognosis of patients with stroke, our study had several limitations. First, this was an observational cohort study from a single‐center hospital. Second, the dynamics of lymphocyte subsets throughout the stroke process were not adequately captured because each patient represented a single data point rather than multiple samples. In addition, in this study, toast typing was performed with a small number of patients with cardioembolic stroke, and therefore this type of data was combined with patients with other stroke types in order to obtain more reliable results from the available data. We plan to expand the sample size in future studies. This will allow us to more accurately analyze and present the characteristics and impact of cardioembolic stroke. Finally, extensive and long‐term prospective clinical studies are necessary to better understand the connection between peripheral lymphocyte subsets and stroke prognosis. Exploring biomarkers for long‐term prognosis could reveal new immunotherapeutic targets for ischemic stroke.

## 5 CONCLUSION

We found that total T‐cell percentage, CD3^+^ T‐cell, and CD4^+^ T‐cell counts were prognostically relevant variables in patients with AIS. Furthermore, a prognosis model based on CD4^+^ T‐cell count showed good accuracy and potential for predicting disease outcomes.

## AUTHOR CONTRIBUTIONS

The study was designed by XS, with material preparation, data collection, and analysis conducted by XZ, SX, and X‐KS. The initial manuscript draft was written by XZ, and subsequent revisions were made by Z‐NG, W‐YD, Y‐NG, and YQ. All authors read and approved the final manuscript. All authors read and reproved the final manuscript.

## FUNDING INFORMATION

This project was funded by the Science and Technology Department of Jilin Province (grant number YDZJ202301ZYTS520) for the benefit of XS.

## CONFLICT OF INTEREST STATEMENT

The authors declare that they have no competing interests.

## Data Availability

The datasets used and/or analyzed during the current study are available from the corresponding author on reasonable request.
